# Tailings Utilization and Zinc Extraction Based on Mechanochemical Activation

**DOI:** 10.3390/ma16020726

**Published:** 2023-01-11

**Authors:** Vladimir I. Golik, Roman V. Klyuev, Nikita V. Martyushev, Vladimir Brigida, Egor A. Efremenkov, Svetlana N. Sorokova, Qi Mengxu

**Affiliations:** 1 Department “Technique and Technology of Mining and Oil and Gas Production”, Moscow Polytechnic University, 38, B. Semenovskaya St., Moscow 107023, Russia; 2 Department of Materials Science, Tomsk Polytechnic University, 30, Lenin Ave., Tomsk 634050, Russia; 3 Federal Research Centre the Subtropical Scientific Centre, Russian Academy of Sciences, 2/28, Yana Fabritsiusa St., Sochi 354002, Russia; 4 Department of Biomedical, Veterinary and Ecological Directions, Peoples’ Friendship University of Russia (RUDN University), 6, Miklukho-Maklaya St., Moscow 117198, Russia; 5 Department of Mechanical Engineering, Tomsk Polytechnic University, 30, Lenin Ave., Tomsk 634050, Russia

**Keywords:** ore, stable mining, mechanical activation, enrichment tailings, zinc leaching

## Abstract

The significant containment of the global mining industry is caused by the problem of the transition to sustainable metal extraction and the integrated use of technogenic raw materials from the tailings of ore processing. The modeling of metal leaching processes using mechanical activation of polymetallic raw material components is particularly important in expanding the application of mining tailings as inert fillers of filling mixtures. This study is aimed at detecting the rotor speed factor on the chemical and mechanochemical effect of zinc yield growth from polymetallic tailings of the mining industry. In this regard, the purpose of this study was to improve the modeling of metal leaching processes using mechanical activation by improving the compositions of the filling mixtures. The methodology of the work included several comprehensive studies: the mechanical activation of tailings during zinc leaching from pulp in the DESI-11 disintegrator; the activation of enrichment tailings and the formation of a filling mass with different parameters of the component composition; the curing of cubic samples and their testing on the IP-1250 press. The Vi Improved text editor was used to prepare the algorithms for deterministic methods of three-dimensional interpolation in the Python language. The experimental results were graphically displayed using Gnuplot. The study of the agitation leaching of the waste obtained from the Sadonskiy mining district results in the fact that the NaCl mass concentration decreased from 13 to 1% and the H_2_SO_4_ concentration stabilization within 0.5 to 0.6% led to a 3-time increase in the zinc yield from the pulp, according to the polynomial law (from 28 to 91%). The obtained results expand the idea of the mechanism of the strength gain by the filling mass under mechanical activation on the components of the filling mixture, as well as changes in the efficiency of zinc leaching at different ratios of two types of lixiviants (sulphuric acid and sodium chloride) in the leaching solution.

## 1. Introduction

The problems of the complex use of tailings that are a technogenic deposit of polymetallic raw materials are especially acute for the world mining industry [1,2,3]. According to Chinese studies, one ton of processed Pb/Zn ore accounts for 0.26 to 2.5 tons of tailings [4,5]. The technologies of metal extraction from the accumulated tailings of the lead-zinc industries often have low efficiency due to the fact that they do not reduce the content of metals to the level of sanitary requirements in various geologic environments [6,7]. In other countries, extensive research is being conducted in the direction of assessing the geochemical properties of contaminated soils [8] (Canada), the peculiarities of the distribution of heavy metals depending on the distance from pollution sources (Nigeria) [9], as well as of the justification of the ways to dispose of zinc waste during road construction [10].

The Sadonskiy mining district (Republic of North Ossetia-Alania) is one of the oldest in Russia. The profitable part of the active balances of the Zgidskoye, Sadonskoye, and Arkhonskoye deposits of the Sadonskaya group amounts to 500.0 thousand tons, 13.0 thousand tons of lead, 24.0 thousand tons of zinc (according to the sum of categories B + C1 + C2). At the same time, ores are divided into substantially lead, lead-zinc and substantially zinc. Quartz-polymetallic ores are related to easily and medium-beneficiated, whereas pyrite–polymetallic ores are related to resistant. Dating back to the 1970s, raw materials from all the above-described fields began to be brought to the Mizurskaya beneficiating plant, while the capacity of ore-mining and processing limit reached 745 thousand tons/g. The ores are processed according to the flotation scheme; as a result, the lead concentrate of the KS-5 brand and the zinc concentrate of the KTs–4 brand are produced. The extraction of metals during beneficiation is as follows: lead is 80–82%; zinc is 82–84%; silver is 60%; cadmium is 56%; bismuth is 32%. The rest of the useful components are lost in the tailings of beneficiation. The resulting wastes are fed to the tailings dump, located 9 km from the plant in the floodplain of the Ardon River. The volume of the geomaterials, accumulated to date in the tailing dump, is approximately four-million tons. As a result, extensive (whose area exceeds 39.4 km^2^) halos of chemical pollution of soils, watercourses and their sediments have been formed.

The transition to the development of copper and impregnated ores due to the development of rich ore-bearing horizons is determined by the growth of preparatory work, the volume of geomaterials lifted to the surface per unit of finished product, as well as the problems of the rock pressure control during the development of reserves in deep horizons [11]. The growth of the global economy and the consumption of rare-earth metals has caused the need to increase the extraction of georesources during the ecological modernization of underground geotechnologies with the widespread use of filling [12,13]. For example, Hua Na proved the possibility of using copper-nickel slag to form expanding filling mixtures with the addition of hexadecyltrimethyl ammonium bromide and Sodium silicate [14]. Moreover, the issues of the geotechnical and technological parameters of the filling mass are far from being improved [15,16]. Hence, the transition to rock formation recycling during metal extraction and the integrated use of technogenic raw materials from tailings remains a fundamental scientific problem. 

At present, the modeling of leaching kinetics is primarily focused on the models of exponential functions and partial differential equations [17,18]. At the same time, both traditional [19,20,21] and conceptually new technologies are being improved: bioleaching [22], phytomining, solvometallurgy [23] or microwave exposure [24]. One of the new directions that has proven its effectiveness (Ceyield reaches 97%) is the selective leaching of geomaterials based on calcination (400 °C) with the addition of sulfur [25]. The chemical and mechanochemical activation of tailings using sulphuric acid is a promising area of improvement in ore processing for the growth of non-ferrous metals production [26,27,28,29,30]. Many studies have proven its efficiency in increasing the kinetics of metal leaching [31,32]. The most modern method for the fine grinding of geomaterials by mechanochemical activation (high-energy ball milling) is high-speed grinding in impact-centrifugal mills, for example, the VSI mill (disintegrators). High-energy grinding absorbs the mixture components with further formation of fine powders, which accelerates the metal leaching processes [31]; at the same time, the iron yield can be increased by 30% [32]. In addition, the leaching efficiency of metals is significantly determined by the ratio of concentrations of various types of lixiviants (for example, H_2_SO_4_ and HCl in [33]). Chalcopyrite grinding into a pin type vertical stirred mill leads to the fact that the Cu leaching kinetics (in terms of crystallinity) is 30% greater when treated with sulfuric acid than that treated with HCL (increase in the dissolution rate = 35 vs 25%). This confirms that the ratio of the concentrations of the two-component solutions on the final yield of polymetallic raw materials is an important factor, the influence of which is not fully understood. 

The purpose of this study is the substantiation of the parameters of the mechanochemical activation of technogenic geomaterials when extracting zinc and utilizing beneficiation tailings of ore mining. In this regard, the study addresses the following tasks: to establish the influence of mechanochemical exposure on the degree of zinc extraction from polymetallic waste; to define the efficiency of the mechanical activation of the filling mixture components based on technogenic wastes. 

## 2. Materials and Methods

To solve the first problem, the object of the study was the technogenic waste of the mining industry, represented by forty-five samples of geomaterial from the tailings of the Mizurskii Mining and Processing Plant (Republic of North Ossetia-Alania). The geomaterial composition of a technogenic deposit is Pb—0.84%, Zn—0.95%, TiO_2_—0.03%, Al_2_O_3_—0.8%, K_2_O—3.5%, Mn—0.015%, Cu—0.18%, Ag—0.015%, S—1.88%, CaO—1.96%, Fe_2_O_3_—4.4%, SiO_2_—31.4%. The Box-Behnken design scheme was implemented in the work in a similar manner [34]. 

**Figure 1:**
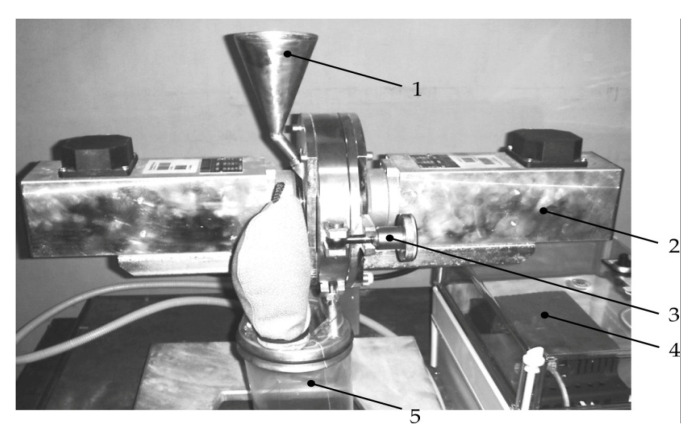
Laboratory device for mechanical activation of geomaterials: 1—hopper for the initial material; 2—disintegrator section with the electric motor; 3—clamping device; 4—control block; 5—storage capacity.

The mechanochemical activation of the pulp was carried out in a DESI-11 disintegrator (Figure 1) with a rotor speed of 50 min^−1^ and 200 min^−1^ (maximum shock rate—up to 240 m/s) for 60 min. The disintegrator consists of two rotors (corfs) rotating in opposite directions, mounted on separate coaxial shafts and enclosed in a casing (whose parts are locked with clamp 3), equipped with electric motors for two spaced-apart sections (2) of the device. Either two or four rows of round cylindrical pins are arranged along concentric circles on the rotor disks so that each row of one rotor freely enters between two rows of the other. The material is fed through a hopper (1) into the central part of the rotor and, moving to the periphery, it is subjected to multiple blows of pins rotating in opposite directions. The processed geomaterials were collected in container 5. The disintegration exposure time was selected based on the available experience, as well as experiments [35,36] on the enrichment of nickel, iron and cobalt. The work [37] found that during ore treatment with 20% sulfuric acid solution, from minute 60 to minute 120 min of enrichment, the Ni yield changed from 88% to 98%, Co—from 96% to 98%, and Fe—from 82% to 90%, which indicates the significance of the first hour of treatment to obtain Zn from the tailings.

Initially, the total weight of 1 L of leaching solution should be set. The program of experiments involved varying the NaCl and H_2_SO_4_ content as follows: h(H_2_SO_4_) = (2, 6 and 10 g/L); h(NaCl) = (20, 90 and 160 g/L). Therefore, 1 L of the standard solution was formed separately for each variant. The volume of water in the solution for each variant was determined as follows:

(1) $$V_{H_{2}O}=1000-V_{H_{2}{\mathrm{SO}}_{4}}-V_{\mathrm{NaCl}},\;\mathrm{mL}$$

where $V_{H_{2}{\mathrm{SO}}_{4}}$—volume of acid for the chosen parameters of the composition (based on the acid density of 1.814 g/L), mL; 

$V_{\mathrm{NaCl}}$—volume of chloride for the chosen parameters of the composition (based on chloride density of 2.165 g/L), mL.

Due to the calculations according to Formula (1), the obtained volumes were recalculated by the weight of water *h*(H_2_O) in a 1000 mL solution (based on the water density = 998.2 g/L at 20 °C). The total weight of the 1 L solution was then determined by simple addition:

(2) $$M_{R}=h(H_{2}{\mathrm{SO}}_{4})+h(\mathrm{NaCl})+h(H_{2}O),\;g$$

Based on the above, the liquid fraction (L) was prepared so that the chemical reagents were first poured in separate flasks in the ratio indicated in the experiments (for example, 2 g/L of sulfuric acid and 20 g/L of sodium chloride), and then the reagents were alternately poured into the flask with a volume of water calculated according to Formula (1).

The sample weight of the geomaterials of the studied waste in each experiment was constant—(50 g). According to the experiments, the ratio of solid and liquid fractions (S/L) varied as follows: 1/4; 1/7; 1/10. As the weight of the polymetallic raw materials in each sample (M_S_) = 50 g, the weight of the liquid fractions (leaching solution—M_L_) was 200, 350 and 500 g, respectively. Next, the weights of the reagents in the total weight of the liquid fraction were determined as follows (using sulfuric acid as an example):

(3) $$m_{L}(H_{2}{\mathrm{SO}}_{4})=\frac{h(H_{2}{\mathrm{SO}}_{4})}{M_{R}}\times M_{L},\;g$$

The geomaterials pre-ground in a ball mill and sieved in a 2.0 mm sieve were mixed with the leaching solution to form slurry. The total pulp weight (M_P_) in each case was determined by the simple addition of different M_L_ and constant values of M_S_. An example of the results of the calculations and the plan of the experiments is shown in Table 1. 

It is known that the graphical solution of three-dimensional problems can be projected onto a two-dimensional X-Y plane. The complexity begins with the four-dimensional response space and, in general, several flat projections can be mutually correlated (for example, as for multispectral images in Remote Sensing), but some of the information is irretrievably lost at the same time. The basis of the novelty of formulating our problem is the departure from the similarity invariant factors (g/L) when it is possible to reduce a multidimensional problem to a finite number of three-dimensional ones. The comparison of several three-dimensional projections onto the same projection plane (in case of controlled reduction to the constant of the studied factors) allows for the reliable assessment of the effect of the presence/absence of several simultaneously acting factors. In general terms, zinc (Zn) leaching is a response function from (h (H_2_SO_4_); h (NaCl); g/L; solid fraction mass (ML); presence/absence of the disintegrator, activation time). To reduce this seven-dimensional problem to three three-dimensional problems, we proceeded from the following logic. The last most significant factor for us (disintegrator influence) was introduced into three separate response functions, while the activation and agitation leaching time was assumed to be constant (60 min), thereby removing it from the brackets: (I) without activation; (II) with a rotor speed of 50 min^−1^; (III) with a rotor speed of 200 min^−1^. One of the most difficult issues was reducing the dimension to two inside the brackets. For this purpose, all of the operations in Table 1 were carried out to transfer from volume concentrations in the leached mixture to the mass concentration in the treated pulp (see Table 2). At the same time, the experiment planning (the ratio of volume concentrations of lixivants) was carried out so that the experimental points were dispersed as widely as possible along the H_2_SO_4_ plane, from 0.3 to 0.9%, and along the NaCl plane, from 1 to 13%.

The mass concentration of the leaching solution components in the final pulp was determined by the following formula (using sulfuric acid as an example):

(4) $$m_{P}{(H}_{2}{\mathrm{SO}}_{4})=\frac{m_{L}{(H}_{2}{\mathrm{SO}}_{4})}{M_{P}}\times 100,\;\%$$

At the last stage, the zinc concentration was determined as a result of pulp enrichment for 60 min via: (I) agitation leaching; (II) leaching with mechanical activation in a disintegrator with rotor speed (υ) = 50 min^−1^; (III) with rotor speed = 200 min^−1^.

**Figure 2:**
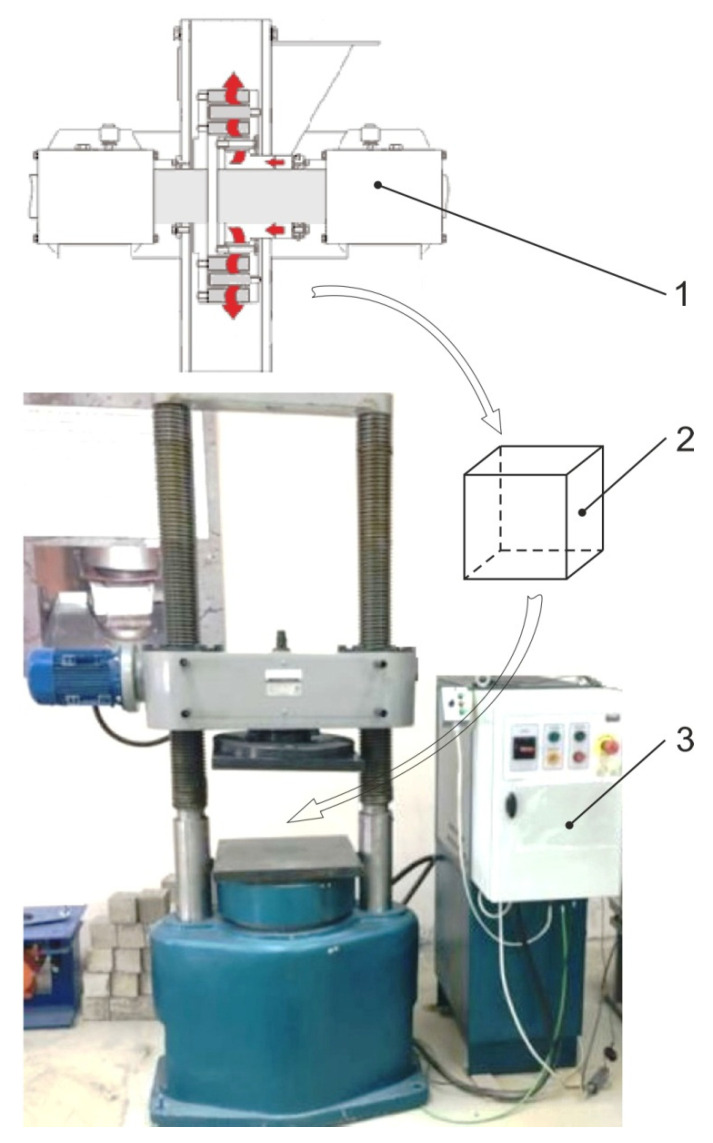
Laboratory bench for sample testing on the IP-1250press: 1—disintegrator; 2—cubic sample; 3—press control unit.

To solve the second problem, we checked the efficiency of the activated tailings as components of the filling mixture (as an inert aggregate). The prepared rock was mixed with cement (M-400) and granulated slag to form a composition with different mixture parameters. The uniaxial compression strength of the filling mixture samples was measured by a standard method (GOST 10180-2012 “Concretes. Strength tests in control samples”). The forms of cubic samples were: 100 × 100 × 100 mm. The experiments on cubic samples aged 3, 7, 28, 60, 90 and 180 days were performed on the IP-1250 machine (ZIPO, Figure 2).

The cubic samples were loaded at a constant rate until geomaterial destruction was achieved. The maximum pressure established during the test was taken as the destructive load in the sample. In accordance with the GOST 18105-86 “Concretes. Strength tests in control samples”, the corresponding algorithm for the reliable adjustment of the obtained results on the base size samples to the resulting values of the filling tensile strength in uniaxial compression (with the number of tests = 5) was chosen.

In most cases, the geostatistical methods for the three-dimensional interpolation of spatial data are performed on the basis of stochastic methods (kriging) [38] in pure form or in combination with machine learning (for example, ANN [39] or random forest method [40]). Thus, the use of “classical” deterministic methods (for example, the k-nearest neighbor method or the finite elements method) is significantly reduced. In this regard, the three-dimensional interpolation was used, according to the operating method, when processing experimental data on the effect of the mechanical activation on the degree of Zn leaching from tailings [41]. The algorithm was implemented as “scripts” in ViIMproved (version 9.0) for the Python language (version 2.7.10). The resulting response surfaces in 3D are obtained using Gnuplot (version 5.4). The regression models were matched by the least square method, using MS Excel. 

## 3. Results

The effect of mechanochemical exposure on zinc extraction from polymetallic waste and the results of the Zn leaching activation using disintegration are shown in Table 2. 

**Figure 3:**
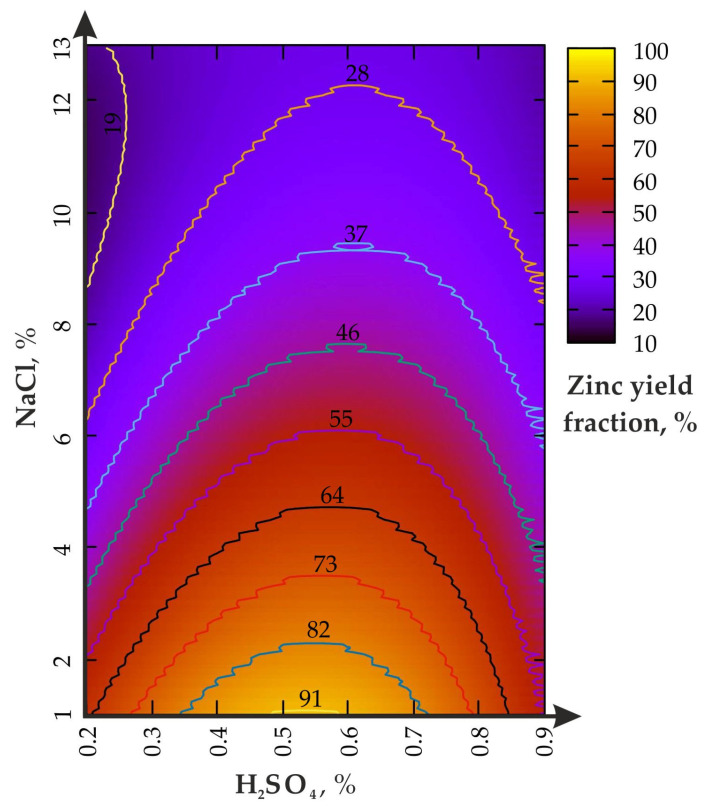
Zinc yield fraction from the enriched geomaterial in the (I) experiment.

The change in the Zn yield in the experiment: (I)—agitation leaching without the disintegrator is shown in Figure 3.

The increase in the H_2_SO_4_ concentration, from 0.2 to 0.9% (Figure 3), activates zinc leaching only at the optimal ratio with NaCl ≤ 1/10. If the NaCl fraction exceeds 6%, then an increase in the H_2_SO_4_ from 0.2 to 0.9% does not lead to the expected effect. The local maximum Zn yield is in the range of 0.5–0.6% for H_2_SO_4_ and 4–1% for NaCl. The excess of the H_2_SO_4_ fraction above 0.7% reduces the Zn concentration from 82 to 55%. The reasons for such phenomenon need further clarification and specification.

During the leaching process at H_2_SO_4_ = 0.9%, the increase in the NaCl fraction, from 1 to 13%, leads to a monotonous decline in the zinc yield, from 55% to slightly less than 28%, which is slightly higher than H_2_SO_4_ = 0.2% (slightly less than 19%). The area of the local maximum (Zn = 82%) is limited to 0.35 to 0.72% for H_2_SO_4_ and from 1 to 2% for NaCl. As a result of the analysis of the data in Table 2, a polynomial dependence (Taylor series) of the zinc extraction rate on the parameters of the leaching solution (R^2^ = 0.96) was established:

(5) $$\begin{array}{c} \mathrm{Zn}=25.7-6.1\mathrm{NaCl}+269.4H_{2}{\mathrm{SO}}_{4}+0.16{\mathrm{NaCl}}^{2}-{222,5H}_{2}{\mathrm{SO}}_{4}{}^{2}-12.0{\mathrm{NaClH}}_{2}{\mathrm{SO}}_{4}+\ldots ,\\ \ldots +0.02{\mathrm{NaCl}}^{3}-44.0H_{2}{\mathrm{SO}}_{4}{}^{3}+17.6{\mathrm{NaClH}}_{2}{\mathrm{SO}}_{4}{}^{2}-0.4{\mathrm{NaCl}}^{2}H_{2}{\mathrm{SO}}_{4}, \end{array}$$

where Zn—zinc yield,%; H_2_SO_4—_mass concentration of sulfuric acid according to pulp weight,%; NaCl—mass concentration of sodium chloride according to pulp weight,%. 

**Figure 4:**
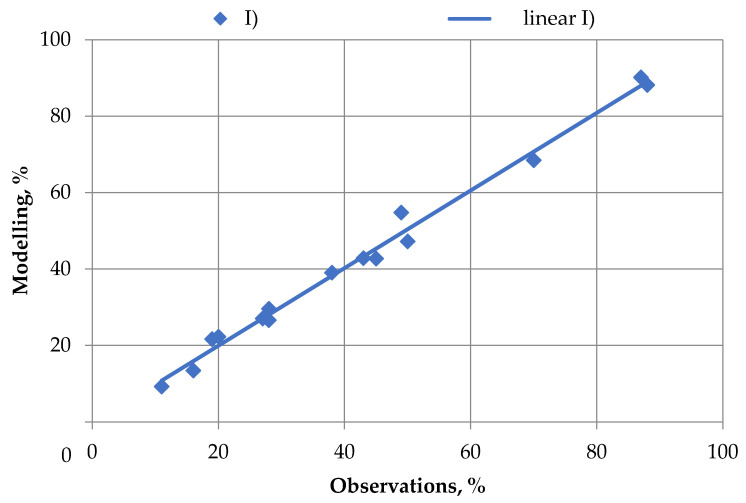
Quality of modeling results (M) with respect to experimental data (E).

The Q-Q graph shown in Figure 4 was the goodness-of-fit criterion to test the quality of the agitation leaching model. 

**Figure 5:**
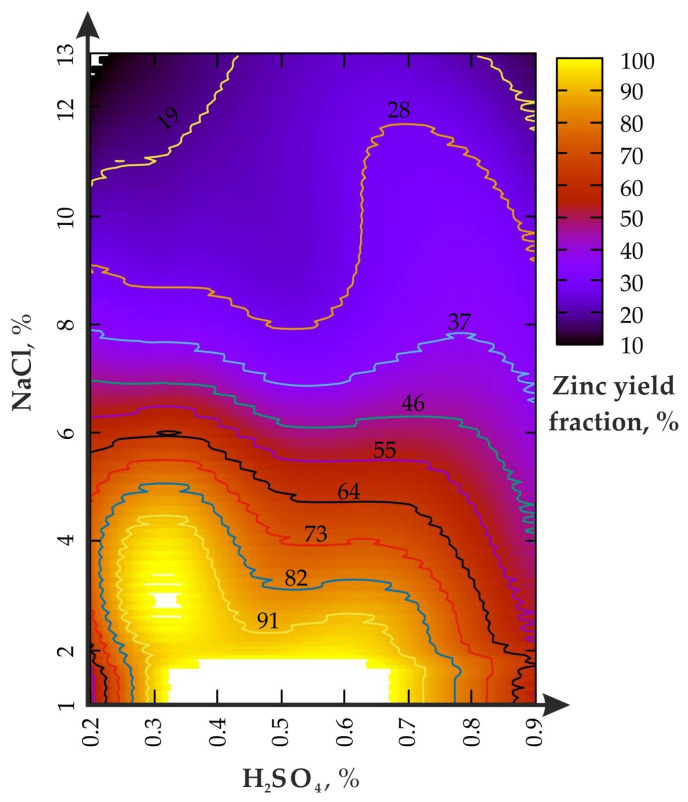
Zinc yield fraction from enriched geomaterial in (II) experiment.

Figure 4 shows that the linear trend for model data is most plausible (R^2^ = 0.98) to the test results. The linear trend equation has the form:

(6) M=1.015O+0.336,

The use of a disintegrator in experiment (II) increases the leaching efficiency as follows (Figure 5). 

The analysis of Figure 5 shows that the chemical-mechanical activation effect of the disintegrator at υ = 50 Hz causes an increase in enrichment productivity in the lower range of the H_2_SO_4_ concentration (0.2–0.4%), while the NaCl concentration should be ≤8%. The H_2_SO_4_ change from 0.2 to 0.8% (NaCl—2–6%) leads to alternating changes in the Zn yield (from 91 to 55%). The increase in the H_2_SO_4_ concentration from 0.2 to 0.9% affects the yield of Zn only at the ratio of NaCl ≤ 1/15. If the NaCl fraction exceeds 8%, the increase in H_2_SO_4_ from 0.2 to 0.9% does not lead to significant changes. The first local maximum of the Zn fraction is in the range of 0.28% for H_2_SO_4_ and 2–4% for NaCl, and the second, from 0.3 to 0.68% for H_2_SO_4_ and 1–1.8% for NaCl.

During leaching at H_2_SO_4_ = 0.9%, the NaCl fraction increases from 1% to 13% and leads to a monotonous decline in the zinc yield, from 65% to slightly less than 19% and less, which is higher than during agitation leaching. The area of the local maximum (Zn of about 100%) is limited by the region, from 0.27 to 0.73% for H_2_SO_4_ and from 1 to 43% for NaCl (when H_2_SO_4_ ≤ 0.4%), as well as from 1 to 2.4% for NaCl (when H_2_SO_4_ is from 0.6 to 0.7%).

**Figure 6:**
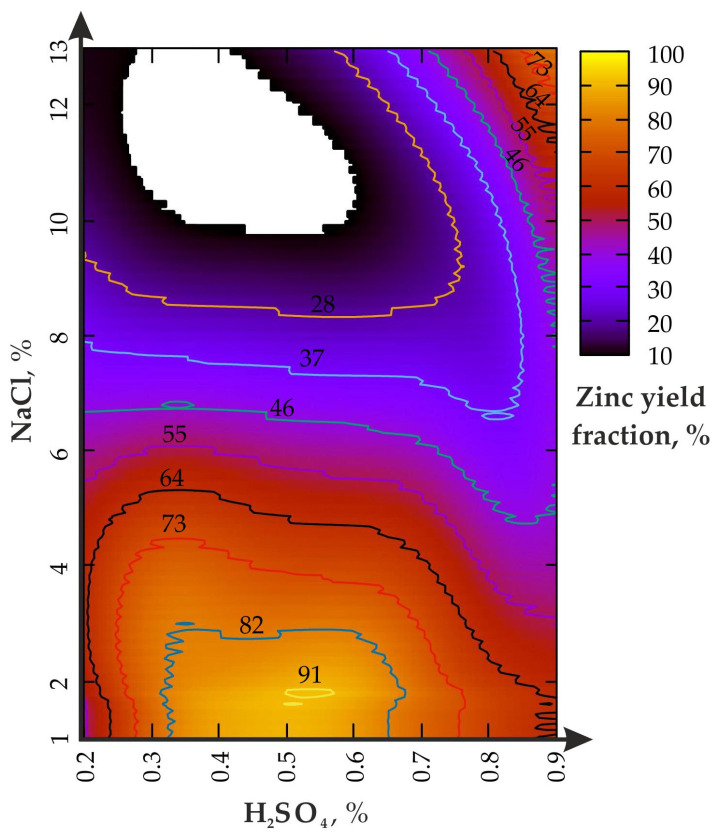
Zinc yield fraction from the enriched geomaterial in the (III) experiment.

The change in the leaching productivity in the III) experiment (under the action of the disintegrator with υ = 200 Hz) is shown in Figure 6.

Figure 6 shows that the chemical-mechanical activation effect of the disintegrator at υ = 200 Hz causes an increase in the enrichment productivity in the upper concentration range of H_2_SO_4_ (0.8–0.9%) and NaCl from 10 to 13%. The H_2_SO_4_ change from 0.2 to 0.8% (NaCl = 10–13%) leads to an increase in the Zn yield (from 10 to 64%). The increase in the H_2_SO_4_ concentration from 0.2 to 0.9% affects the yield of Zn only at NaCl ≤ 8%. The first local maximum of the Zn fraction is in the range between 0.5 and 0.6% for H_2_SO_4_ and 1–2.5% for NaCl; the second is between 0.85 and 0.9% for H_2_SO_4_ and 10–13% for NaCl.

In terms of the mechanical activation of the filling mixture components based on technogenic waste, this problem was solved by the fact that, as a result of solving the first problem, it was revealed that the treatment of geomaterial with a rotor speed of 50 min^−1^ is sufficient for effective activation. In this regard, the dry mixture TsKhSh was subjected to disintegration with a rotor speed of 50 min^−1^ before concrete mixing for at least an hour.

According to the XRD standard measurements of the TERRA Olympus Laboratory (Japan), the chemical composition of the tailings before and after beneficiation changed significantly.

Thereafter, the treated composition was mixed with pure water, according to the standard procedure, and tested in the manner described above. The consumption of the components per 1 m^3^ of the filling mass is shown in Table 3. 

**Figure 7:**
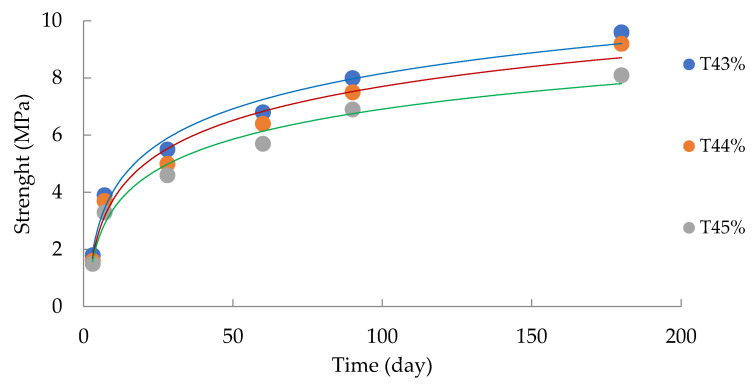
Change in the mixture strength depending on the strength gain duration.

The analysis of the data in Table 4 made it possible to determine the operational characteristics and physical and mechanical parameters of the filling mass (Figure 7). 

The power models of the response function and the dynamics of the tensile strength in the uniaxial compression of the tailings are determined by the following equations:

- for the mass fraction of tailings (t) = 43% (800 kg), the logarithmic dependence of the filling strength on the hardening duration (R^2^ = 0.98) is established:
  (7) $$R_{c}=1.78\ln(t)-0.06,$$
- for the mass fraction of tailings (t) = 44% (835 kg), the logarithmic dependence of the filling strength on the hardening duration (R^2^ = 0.97) is established:
  (8) $$R_{c}=1.71\ln(t)-0.19,$$
- for the mass fraction of tailings (t) = 45% (860 kg), the logarithmic dependence of the bookmark strength on the hardening duration (R^2^ = 0.97) is established:
  (9) $$R_{c}=1.52\ln(t)-0.09,$$
  where R_c_—compressive strength, MPa; t—strength gain duration, days.

The obtained results have been summarized in Table 5.

## 4. Discussion

One of the main results of the work is the confirmation of the main role of sulfuric acid, both for agitation leaching and using the disintegrator. At the same time, the mechanochemical effect on the pulp leads to a multiple increase in the maximum productivity zone (see the area bounded by the 91% curve in Figure 5). Moreover, one of the maxima is shifted to a value of 0.3% for H_2_SO_4_ (versus 0.5–0.6% for agitation leaching) when NaCl = from 2 to 4%, which significantly saves acid consumption. The analysis of the results of the first problem shows the general regularity of sodium chloride operation similar to studies [42,43]. In addition, study [37] revealed the effects of the mechanical processing (from 60 to 480 min^−1^) of nickel ore in the ball mill during the leaching of Ni, Fe and Co. The concentration of H_2_SO_4_ varied between 200 and 400 g/L (at S/L—1/3), while approximately 80% of the total increase in the yield of the polymetallic raw materials was leached in the first 60 min. The difference for the minimum and maximum rotor frequency, in terms of the leaching efficiency, did not exceed 35%, which is confirmed by the use of low-speed mechanical exposure in our studies. In another similar study [44,45], the mechanical activation of the ore in the stirred ball milling was conducted with the rotor RPM from 110 to 428 min^−1^ with subsequent exposure to H_2_SO_4_ to obtain Cu. It was found that in the first 10% of the enrichment time, up to 80% of the productivity of leaching the metal from the pulp was achieved, while the difference in the productivity of Cu separation at low and high rotations did not exceed 42%. The results of the enrichment of bauxitic clay to obtain Li, Al, Fe and Mg during the calcination of the samples (600 °C)m with subsequent exposure to sulfuric acid at the ratio S/L = 1/5, are indirectly confirmed [46]. In general, the parameters of Zn leaching efficiency are confirmed by the results of studies conducted by foreign authors [47,48]. The considered problem was reflected in the works of mining experts [26,27]. 

The mechanism of mechanochemical activation, apparently, may be as follows. The use of the disintegrator to activate the pulp conditions requires the accumulation of energy of a special kind (not thermal) in it. An increase in the temperature of the geomaterials is accompanied by a structural change in its state, when the uncompensated conversion of work into heat becomes impossible. The “compensation” implies a change in the state of the material or mechanical activation. The main reason for this is the overload experienced by the particles in the pulp, which can be up to four hundred million accelerations of free fall.

The analysis of Figure 7 allows for the conclusion that the parameters of long-term strength, in general, meet the standard terms of hardening for PAO GMK Norilsk Nickel (Regulations of Technological Production Processes when conducting stowing operations at the mines of the ZF OAO GMK Norilsk Nickel (RTPP-045-2004), Norilsk—2005—P. 55). Moreover, more than 75% of the compressive strength of the rocks was achieved in the first 28 days of setting. Such logarithmic curves were defined by Francisca Perez-Garcia in [49]; the authors also defined 28 days as the main period of setting. This, on the whole, confirms the correctness of the direction of our research inquiry.

## 5. Conclusions

Sustainable metal mining is a set of measures to minimize the anthropogenic impact of mining and to ensure the enrichment and transportation of polymetallic raw materials to prevent environmental degradation in the future. One of the main results of the study is the substantiation of the parameters of the mechanical-chemical-activation effect on the components of technogenic geomaterials, thus expanding the scope of application of the tailings of the mining industry as inert fillers for filling mixtures. The use of the deterministic method of the three-dimensional interpolation of incomplete scattered data in combination with polynomial regression allowed us to obtain the following results:

- for the first time, during agitation leaching, a decrease in the NaCl mass concentration, from 13 to 1% at the H_2_SO_4_ concentration from 0.5 to 0.6%, has been found to lead to the 3-time Zn yield increase (accompanied by the local maximum area formation (Zn = 91%) from the pulp, according to the polynomial dependence.
- at a low rotation speed of the disintegrator rotors, for the first time, the H_2_SO_4_ concentration decreased from 0.8 to 0.26% at a NaCl concentration from 3 to 4%, and has been established to lead to a Zn yield increase in the pulp, from 64 to 91%. At that, the local maximum area has been shifted towards a lower consumption of sulfuric acid and its area by an order of magnitude is higher than that in the case of agitation leaching.
- the amount of zinc concentration in the mechanically activated pulp is increased from three to four times, with a decrease in the concentration of sodium chloride and an increase in the fraction of sulfuric acid in the leaching solution.
- the peculiarities of the strength gain mechanism by the filling mass caused by the effect of mechanical activation on the components of the mixture composition include the increase in the mass uniaxial compression strength according to the logarithmic law to the required values, and the main increase in the strength characteristics is achieved during the first 28 days of hardening.

The practical significance of the obtained results are that the revealed regularities can be used to substantiate the parameters of the combined mechanochemical activation of metal extraction from the off-grade raw materials of technogenic deposits. The industrial realization of this direction will make it economically attractive to locate beneficiating plants as close as possible to the mines. This will allow the combining of the processes of deep processing of polymetallic raw materials accompanied by the subsequent transportation of tailings through pulp pipelines for the subsequent use as an inert filler when laying the mined-out space. For the conditions of PAO “GMK Norilsk Nickel”, the practical possibility of an ecological transition to the recycling of rocks has been proven. In this case, the concept of sustainable development will be implemented in terms of the absence of dump masses on the surface of the claims of mines, while reducing the cost of managing the mining pressure. 

The presented conclusions are valid only in the cases of considering geomaterials obtained from the deposits of the Sadonskaya group (Zgidskoye, Sadonskoye, Arkhonskoye). The main limitations include: the mechanochemical activation duration of 60 min; the rotation speed of the disintegrator rotors ranging between 50 min^−1^ and 200 min^−1^; the sulfuric acid content in the leaching solution ranging between 2 and 10 g/L; the leaching solution content of sodium chloride ranging from 20 to 160 g/L; the ratio of solid and liquid fractions (S/L) ranging between 1/4 and 1/10. The obtained results determine the need for further research on using disintegrators to increase the metal leaching productivity when using various types of lixiviants (for example, HCl, HNO_3_ or other acids).

## Figures and Tables

**Table 1:** Results of calculations of experimental variants.

N	*h* (H_2_SO_4_)	*h* (NaCl)	*V* (H_2_SO_4_)	*V* (NaCl)	*V* (H_2_O)	*h* (H_2_O)	M_L_	S/L	M_L_	m_L_ (H_2_SO_4_)	m_L_ (NaCl)	M_P_
	g/L	g/L	mL	mL	mL	g/L	g		g	g	g	g
1	2	20	1.1	9.2	989.7	987.9	1009.9	1/4	200	0.40	3.96	250
2	10	20	5.5	9.2	985.3	983.5	1013.5	1/4	200	1.97	3.95	250
3	2	160	1.1	73.9	925.0	923.3	1085.3	1/4	200	0.37	29.48	250
4	10	160	5.5	73.9	920.6	918.9	1088.9	1/4	200	1.84	29.39	250
5	2	20	1.1	9.2	989.7	987.9	1009.9	1/10	500	0.99	9.90	550
6	10	20	5.5	9.2	985.3	983.5	1013.5	1/10	500	4.93	9.87	550
7	2	160	1.1	73.9	925.0	923.3	1085.3	1/10	500	0.92	73.71	550
8	10	160	5.5	73.9	920.6	918.9	1088.9	1/10	500	4.59	73.47	550
9	6	90	3.3	41.6	955.1	953.4	1049.4	1/7	350	2.00	30.02	400
10	6	20	3.3	9.2	987.5	985.7	1011.7	1/4	200	1.19	3.95	250
11	6	160	3.3	73.9	922.8	921.1	1087.1	1/4	200	1.10	29.44	250
12	6	20	3.3	9.2	987.5	985.7	1011.7	1/10	500	2.97	9.88	550
13	6	160	3.3	73.9	922.8	921.1	1087.1	1/10	500	2.76	73.59	550
14	2	90	1.1	41.6	957.3	955.6	1047.6	1/7	350	0.67	30.07	400
15	10	90	5.5	41.6	952.9	951.2	1051.2	1/7	350	3.33	29.97	400

**Table 2:** Extraction of zinc from polymetallic waste.

N	m_P_ (H_2_SO_4_)	m_P_ (NaCl)	Experiment		
	%	%	(I)	(II)	(III)
1	0.16	1.58	45	27	32
2	0.79	1.58	70	79	61
3	0.15	11.79	11	11	13
4	0.73	11.75	28	28	27
5	0.18	1.80	49	47	42
6	0.90	1.79	50	55	53
7	0.17	13.40	16	6	12
8	0.83	13.36	19	16	65
9	0.50	7.50	43	32	27
10	0.47	1.58	87	97	81
11	0.44	11.77	28	21	7
12	0.54	1.80	88	99	82
13	0.50	13.38	27	21	15
14	0.17	7.52	20	40	31
15	0.83	7.49	38	38	23

**Table 3:** Filling strength when changing the tailings fraction.

N	Concrete	Tailings	Slag	Water	Strength Gain Duration, Days					
	kg	kg	kg	kg	3	7	28	60	90	180
1	130	800	450	400–450	1.8	3.9	5.5	6.8	8	9.6
2	115	835	430	400–450	1.6	3.7	5	6.4	7.5	9.2
3	100	860	420	400–450	1.5	3.3	4.6	5.7	6.9	8.1

**Table 4:** Parameters of geomaterials before (I) and after (II) zinc leaching.

	Pb	Zn	TiO_2_	Al_2_O_3_	K_2_O	Mn	Cu	Ag	S	CaO	Fe_2_O_3_	SiO_2_
I	0.84	0.95	0.03	0.80	3.50	0.02	0.18	0.02	1.88	1.96	4.40	31.40
II	0.35	0.51	0.02	0.56	2.63	0.01	0.14	0.01	1.32	1.31	3.96	21.98

**Table 5:** Generalized parameters of the strength gain for different compositions of the mixture.

T, %	Formula	R^2^
43	$R_{c}=1.78\ln\left(t\right)-0.06$	0.98
44	$R_{c}=1.71\ln(t)-0.19$	0.97
45	$R_{c}=1.52\ln(t)-0.09$	0.97

## Data Availability

The data presented in this study are available from the corresponding authors upon reasonable request.
